# Environmental factors for establishment of the dormant state in oocytes

**DOI:** 10.1111/dgd.12653

**Published:** 2020-02-27

**Authors:** Katsuhiko Hayashi, So Shimamoto, Go Nagamatsu

**Affiliations:** ^1^ Department of Stem Cell Biology and Medicine Graduate School of Medical Sciences Kyushu University Fukuoka Japan

**Keywords:** follicle, germ cell, oocyte

## Abstract

Guaranteeing the sustainability of gametogenesis is a fundamental issue for perpetuating the species. In the mammalian ovary, sustainability is accomplished by keeping a number of oocytes “stocked” in the dormant state. Despite the evident importance of this state, the mechanisms underlying the oocyte dormancy are not fully understood, although it is presumed that both intrinsic and extrinsic factors are involved. Here, we review environmental factors involved in the regulation of oocyte dormancy. Consideration of the environmental factors illustrates the nature of the ovarian compartment, in which primordial follicles reside. This should greatly improve our understanding of the mechanisms and also assist in reconstitution of the dormant state in culture. Accumulating knowledge on the dormant state of oocytes will contribute to a wide range of research in fields such as developmental biology, reproductive biology and regenerative medicine.

## Graphical abstract

A number of oocytes are in a dormant state under a specific condition in the mammalian ovary. This article reviews environmental factors that establish the dormancy of the oocytes.

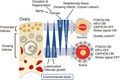

## 1 INTRODUCTION

**Figure 1:**
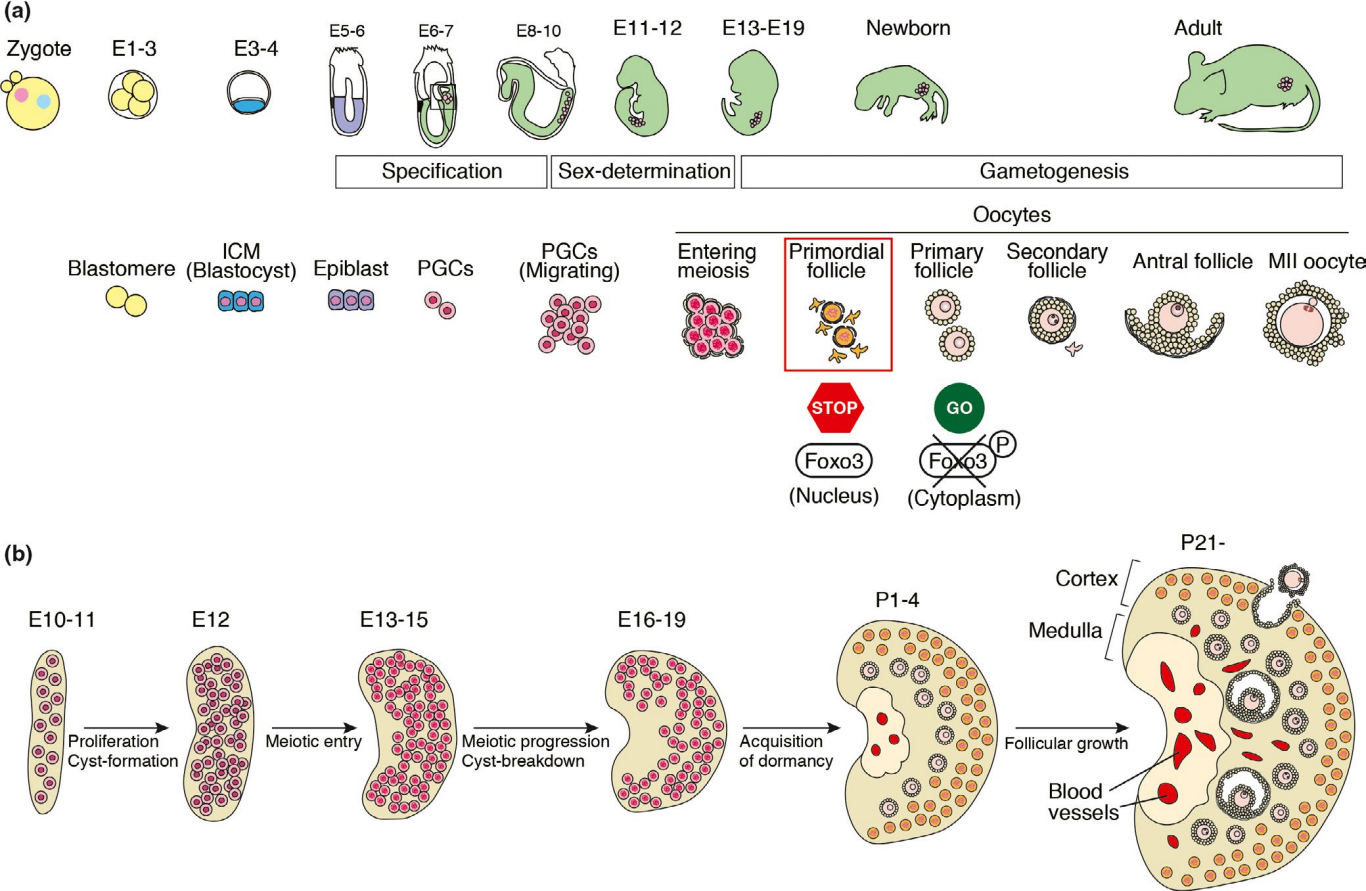
Germ cell development and ovarian organogenesis in mice. (a) A schematic diagram of germ cell development. In female, PGCs enter meiosis after reaching the ovary, thereby becoming oocytes. Whether oocytes stay dormant or start growing is regulated by subcellular localization of FOXO3. FOXO3 is localized in the nucleus of the dormant oocyte. Upon phosphorylation via a PI3K‐mediated signaling pathway, FOXO3 is transported to the cytoplasm and degraded, thereby starting oocyte growth. (b) Ovarian organogenesis and distribution of oocytes. At the perinatal stage, dormant oocytes are localized at the cortical region. The distribution is maintained in the adult ovary

Germ cell lineage is the sole lineage that can transmit genetic and epigenetic information to the next generation. In mammals, development of the lineage can be divided into three periods: specification, sex‐differentiation and gametogenesis (Figure 1a). Specification is the stage in which a founder population of the germ cell lineage, named primordial germ cells (PGCs), is specified from the pluripotent cell population. In mammals, this occurs at a quite early stage of embryogenesis, typically around gastrulation (Lawson & Hage, 1994; Ohinata et al.., 2005). PGCs are formed in an extremely posterior part of the embryo and then migrate into the genital ridges, which are the future ovaries or testes. Sex‐determination occurs in the gonads. In response to the signals provided by somatic cells of the ovary or the testis, PGCs differentiate in a sex‐dependent manner (Jameson et al., 2012; McLaren, 2000). PGCs enter meiosis in the ovary, thereby becoming oocytes, whereas PGCs arrest their cell cycle at G1 in the testis to become prospermatogonia. The number of oocytes peaks at this stage, since oocytes are no longer proliferative, whereas prospermatogonia still possess the potential to proliferate. In fetal ovaries, the oocytes are connected to each other through intercellular bridges that are formed by incomplete cytokinesis during proliferation of PGCs in the gonad (Figure 1a). In the perinatal stage the bridges are broken via a process called cyst‐breakdown, and many oocytes are eliminated from the ovary by cell death. The remaining oocytes form primordial follicles, in which individual oocytes are enclosed by squamous granulosa cells (Figure 1a). In the testis, prospermatogonia resume mitosis in the postpartum stage to become spermatogonial stem cells that support production of a robust number of sperm during throughout nearly the entire life cycle. Gametogenesis is therefore dimorphic from the beginning: oogenesis starts from dormant oocytes that arrest at a diplotene stage of meiosis prophase I in the ovary, whereas spermatogenesis starts from spermatogonial stem cells that are still in mitosis in the testis.

The most immature oocytes in the ovary are encapsulated in primordial follicles. Histologically, a primordial follicle is composed of an oocyte surrounded by a monolayer of squamous granulosa cells. In many species, primordial follicles tend to locate at a cortical region of the ovary (Figure 1b). This localization suggests that the local environment would be important for maintenance of the dormant state, although the contributions of this environment are not fully understood. Genetic studies using knockout mice partially uncovered the oocyte‐intrinsic regulatory mechanism for maintenance of the dormant state. *Foxo3*, a member of the Fox gene group O, is known to play a central role in the maintenance (Figure 1a). *Foxo3*‐disrupted mice quickly lose their fertility due to the overactivation of immature follicles, resulting in premature ovarian failure (Castrillon, Miao, Kollipara, Horner, & Depinho, 2003). On the other hand, overexpression of *Foxo3* makes oocytes refractory to growth (Liu et al., 2007). FOXO3 is regulated by phosphorylation via a phosphatidylinositol‐3 kinase (PI3K)‐mediated signaling pathway. Upon its phosphorylation, FOXO3 is transported from the nucleus to the cytoplasm, thereby evoking oocyte growth (Figure 1a). Disruption of *Pten*, a negative regulator of PI3K signaling, also leads to an overactivation of follicles similar to that in *Foxo3* knockout mice (Reddy et al., 2008). Moreover, the addition of a PI3K‐inhibitor, LY294002, to organ culture of ovaries inhibits oocyte growth (Nagamatsu, Shimamoto, Hamazaki, Nishimura, & Hayashi, 2019). These findings reinforce the notion that PI3K signaling is involved in maintenance of the dormant state. As an extrinsic factor to activate the PI3K signaling, stem cell factor (SCF) has been proposed. SCF and its receptor, c‐kit, are expressed in granulosa cells and oocytes, respectively, in primordial follicles. Activation of the signaling pathway is important for exiting the dormant state and triggering oocyte growth (Zhang et al., 2014). Although the signaling pathway in oocytes has not been fully addressed at the level of biochemistry, PI3K is known to be one of the downstream factors of c‐kit in other cell lineages. Although these signals control the balance between the dormant and active state, it is still unclear how they are locally controlled in the ovary. In this review, we discuss environmental factor(s) that contribute to the dormant oocytes in the ovary.

## 2 IDENTIFICATION OF THE GENE EXPRESSION PROFILE OF DORMANT OOCYTES

**Figure 2:**
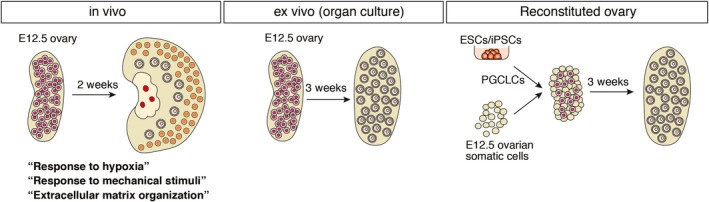
Synchronous follicular development in culture. A large number of primordial follicles are formed at the perinatal stage (left). In contrast, very little primordial follicle was formed in organ culture of fetal ovaries (middle) and reconstituted ovaries (right). Gene ontology analysis of gene expression enriched in dormant oocytes in vivo showed the following terms: “Response to hypoxia”, “Response to mechanical stimulus” and “Extracellular matrix organization”

A clue to identifying the environmental factors involved in oocyte dormancy was revealed during the transplantation of fetal ovaries into adult mice. In the fetal ovaries transplanted into the kidney capsule or the ovarian bursa of the adult mice, there was very little primordial follicle formation at 4–5 weeks after transplantation (Hayashi et al., 2012; Matoba & Ogura, 2011). This was also the case in in vitro culture of fetal ovaries (Morohaku et al., 2016): most of the oocytes in the culture grew simultaneously and few oocytes remained dormant (Figure 2). These studies indicate that the environmental conditions necessary for the maintenance of dormancy are not yet established in the fetal ovaries.

In parallel, we established a culture system to produce functional oocytes from pluripotent stem cells, such as embryonic stem cells (ESCs) and induced pluripotent stem cells (iPSCs) (Hayashi, Hikabe, Obata, & Hirao, 2017; Hikabe et al., 2016). In this culture system, ES cell‐derived PGC‐like cells (PGCLCs) were aggregated with somatic cells from E12.5 fetal ovaries, followed by in vitro culture. We found that PGCLCs consistently grew to mature oocytes in a synchronous manner and few oocytes remained dormant (Figure 2). Since this culture system makes it easier to collect large numbers of oocytes, we were able to use it compare the gene expression profiles during oocyte differentiation in vivo and in vitro, which should identify genes specifically expressed in dormant oocytes.

Comparison of the gene expression profiles during oocyte differentiation in vivo and in vitro illustrated the dormant state of the oocytes (Shimamoto et al., 2019). Consistent with its functional requirement, Foxo3 was highly expressed in the dormant oocytes. Interestingly, genes involved in the reduction of reactive oxygen species (ROS) were also enriched in this population, suggesting that the dormant state protects oocyte from oxidation. This is rational, since dormant oocytes are kept for a long time in the ovary, perhaps decades in humans. Since Foxo3 plays a central role in the dormancy, we further investigated genes whose expression was changed upon enforced expression of Foxo3 in the oocytes in the reconstituted ovary. Gene ontology analysis of such genes yielded the GO terms “response to hypoxia”, “response to mechanical stimulus” and “extracellular matrix organization”, which seem to be relevant to environmental factors in the ovary (Figure 2) (Shimamoto et al., 2019).

## 3 IMPACT OF HYPOXIA ON DORMANCY OF THE OOCYTES

**Figure 3:**
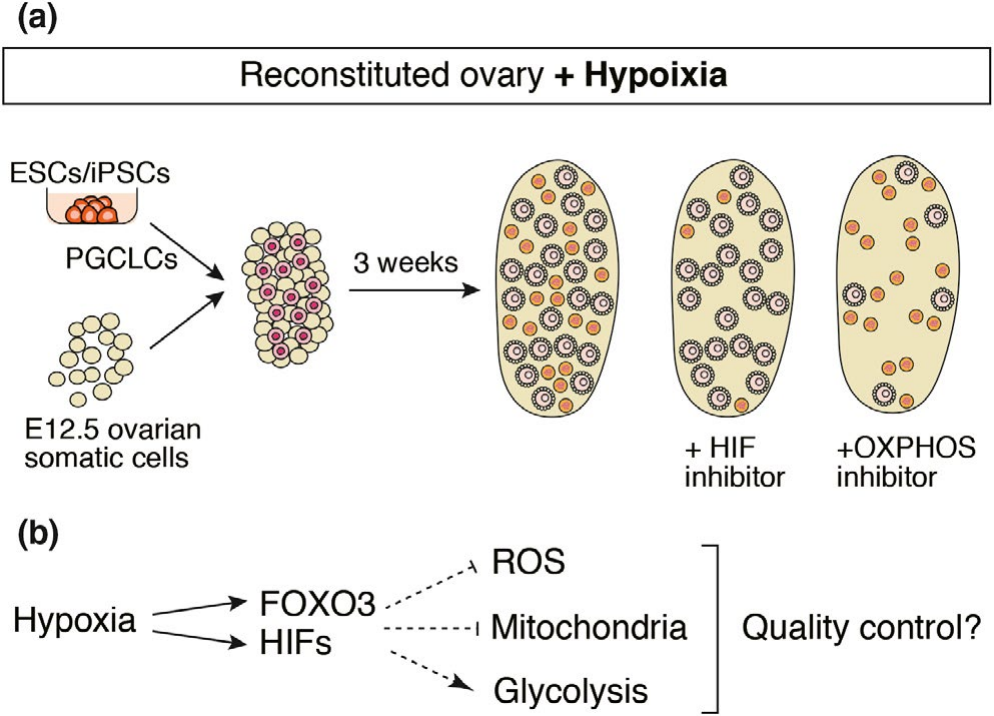
Hypoxia contributes to establishment of the dormant state. (a) Follicle formation under a hypoxic condition. A number of primordial follicles were formed in ovaries cultured under a hypoxic condition. In culture with YC1, a HIF inhibitor, primordial follicles were selectively eliminated. In contrast, in culture with rotenone, an OXPHOS inhibitor, large follicles were selectively eliminated, whereas primordial follicles were relatively unaffected. (b) A putative regulatory network in dormant oocytes. Hypoxia induces expression of FOXO3 and HIF proteins. These transcription factors control ROS, nuclear‐encoded mitochondrial genes, and glycolysis

The effects of hypoxia on oocyte dormancy were tested by culturing reconstituted ovaries under a hypoxic condition (5%O_2_, 7%CO_2_ and 90%N_2_). The results were clear, as the number of small oocytes was drastically increased in the reconstituted ovary under this condition (Figure 3a). In addition, FOXO3 was localized in the nuclei of the small oocytes, suggesting that the hypoxia‐induced small oocytes were dormant. The results of transcriptome analysis of the oocytes under the same hypoxic condition were similar, but not identical, to the results for oocytes in the primordial follicles in vivo (Shimamoto et al., 2019). This fact indicates that another factor is involved in the establishment and/or maintenance of the dormant state in oocytes. Although it is difficult to measure the exact oxygen concentration in a specific part of the ovary, it is known that there are relatively few blood vessels in the cortical region of the ovary. This may be consistent with the effect of hypoxia observed in the culture system.

Hypoxia‐induced factor (HIF) proteins, known as a downstream of hypoxia, are involved in dormancy of the oocytes. Both Hif1α and Hif2 α are expressed in oocytes in the primordial follicles. Although disruption of these genes in dormant oocytes has not been reported, culture experiments showed that inhibition of HIF1A and HIF2A by YC1 in reconstituted ovaries selectively eliminated small oocytes under a hypoxic condition (Figure 3a) (Shimamoto et al., 2019). HIFs are known to change metabolic pathways by promoting anaerobic glycolysis and inhibiting oxidative phosphorylation (OXPHOS). Consistently, the addition of rotenone, an inhibitor of OXPHOS, to a culture of reconstituted ovaries under the hypoxic condition eliminated large oocytes, whereas small oocytes were relatively unaffected (Figure 3a) (Shimamoto et al., 2019). These findings suggested that hypoxia activates HIFs that endow the small oocytes with independency of OXPHOS. Interestingly, *Foxo3* is a target of HIF1A and antagonizes OXPHOS through repression of a set of nuclear‐encoded mitochondrial genes (Jensen et al., 2011). Taking these findings together, we consider that hypoxia may orchestrate the dormant state of oocytes through activation of HIFs and FOXO3 in the ovary (Figure 3b). It is widely known that HIFs are involved in the reduction of ROS (Stegen et al., 2016; Zhao et al., 2014). Detoxification of ROS in oocytes is particularly important for maintaining the quality of oocytes in the presence of oxidative stress. Metabolic control by HIFs and FOXO3 could be a key molecular network involved in the maintenance of quality of oocytes for a long period of time in the ovary.

## 4 EXTRACELLULAR MATRIX EXERTS MECHANICAL STRESS ON DORMANT OOCYTES

**Figure 4:**
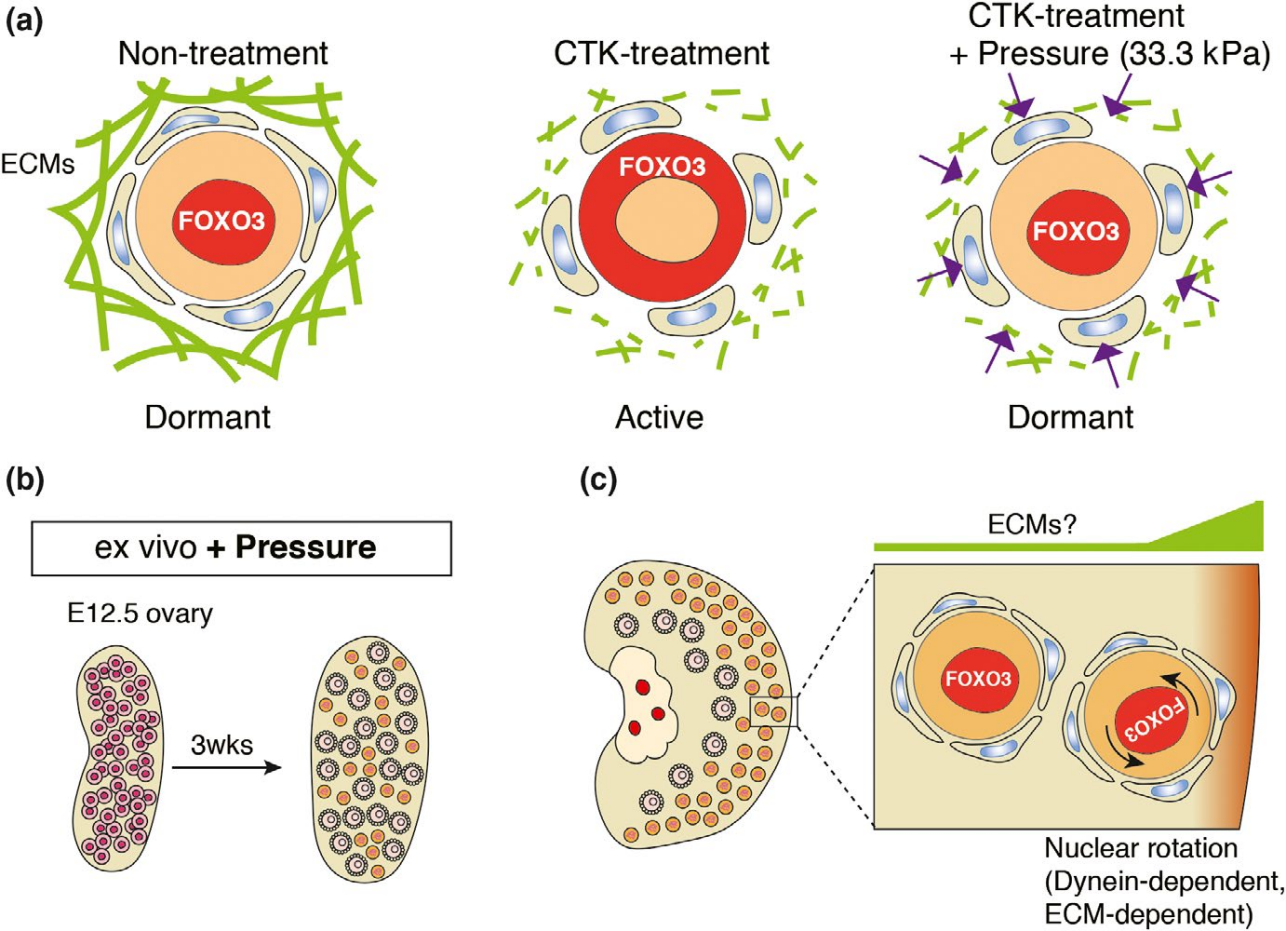
Mechanical stress contributes to establishment of the dormant state. (a) Localization of FOXO3 in response to mechanical stress. In the dormant oocyte, FOXO3 is localized in the nucleus (left). Upon digestion of ECMs, FOXO3 was translocated into the cytoplasm (middle) and was restored in the nucleus with additional air pressure. (b) Primordial follicle formation in the organ‐culture with additional air pressure. (c) Nuclear rotation in the oocytes. The nuclei of oocytes in primordial follicles at the edge of the ovaries rotate, whereas those inside the ovaries are stationary. This difference may be due to a different concentration of ECMs in the ovary. The nuclear rotation ceased in the ovary treated with collagenase or dynein inhibitor

As described above, the GO analysis of genes regulated by FOXO3 revealed the possible involvement of extracellular matrix (ECM). Histologically, ECM is enriched in a cortical region of the ovary, where primordial follicles reside (Bochner, Fellus‐Alyagor, Kalchenko, Shinar, & Neeman, 2015). When whole ovaries were incubated with a collagenase‐containing solution that digests ECM at the cortical region, dormancy of the oocytes was broken. In collagenase‐treated ovaries, translocation of FOXO3 into cytoplasm was observed in the oocytes at the edge of the cortical region, and some of the oocytes indeed started follicular growth to secondary follicles (Figure 4a) (Nagamatsu et al., 2019).

Cells recognize ECM though integrin molecules on their surface. The adherent signals and mechanical stress are transduced though focal adhesions that connect to polymerized actin filaments, named stress fibers. The focal adhesions and stress fibers are highly dynamic, and depend on the quantity and quality of the ECM. It is worth noting that the cell shape is well correlated with the concentration of ECM: the higher the concentration of ECM in the cells, the flatter the cell shape will be (Smith, Cho, & Discher, 2017). In addition, it is known that such morphological changes in cell shape are accompanied by changes in gene expression and differentiation capacity in the cells (Engler, Sen, Sweeney, & Discher, 2006). This association might be relevant to the histological features of squamous granulosa cell primordial follicles, since the first sign of follicular development is change in the shape of granulosa cells from squamous to cuboidal. In the primordial follicles, stress fibers are observed in both oocytes and squamous granulosa cells (Nagamatsu et al., 2019). Our experiments showed that granulosa cells of the primordial follicles became cuboidal upon collagenase treatment. Consistent with the morphological change, stress fibers became faint upon digestion of ECM (Nagamatsu et al., 2019).

Based on the morphological features, what kinds of mechanical force are exerted on the primordial follicles? Because it has been proposed that flat cells tend to have a high surface tension (Lecuit & Lenne, 2007), squamous granulosa cells with stress fibers would have a higher surface tension than cuboidal granulosa cells. In their tensed state, the granulosa cells would confer compression stress to the oocytes. In this way, compression could be a key factor for maintaining dormancy in the oocytes. This hypothesis seems to have been confirmed in our study, since the number of dormant oocytes was increased in collagenase‐treated ovaries cultured in a high air pressure chamber (Figure 4a) (Nagamatsu et al., 2019). In the chamber, 33.3 kPa in addition to atmospheric pressure was supplied to the tissue. Under this condition, the number of oocytes with nuclear FOXO3 in the collagenase‐treated ovaries reverted to the number in non‐treated ovaries. Moreover, culturing E12.5 fetal ovaries with the additional air pressure resulted in a number of small oocytes with nuclear FOXO3 (Figure 4b). These observations indicate that mechanical stress is a requirement for establishing and/or maintaining the dormancy in the oocytes, though the mechanisms transducing the mechanical signal are not clear.

## 5 NUCLEAR ROTATION IN OOCYTES IN PRIMORDIAL FOLLICLES

Mechanical stresses result in various cellular responses. One of the outcomes is rotation of the nucleus. Although the biological significance of nuclear rotation is still elusive, it has been reported that the rotation is coupled with a mechanical stress in myotubes (Wilson & Holzbaur, 2012), neurons (Wu, Umeshima, Kurisu, & Kengaku, 2018), fibroblasts (Levy & Holzbaur, 2008), and cell lines (Brosig, Ferralli, Gelman, Chiquet, & Chiquet‐Ehrismann, 2010; Gerashchenko, Chernoivanenko, Moldaver, & Minin, 2009; Paddock & Albrecht‐Buehler, 1988). For example, in the above‐mentioned studies on neurons and fibroblasts, cell‐stretching during cell migration triggered nuclear rotation. Such a nuclear rotation is driven by microtubules associated with motor proteins. It is known that the nuclear membrane is anchored by the linker of nucleoskeleton and cytoskeleton (LINC) complex that connects to the cytoskeletons via motor proteins (Crisp et al., 2006). The cytoskeletons are also connected to the cell membrane, so that a mechanical stress sensed at the cell membrane can be transduced to the nucleus. Since the nuclear membrane is pulled by motor proteins associated with microtubules, nuclear rotation is likely to occur at unbalanced point forces caused by conformational change in response to a mechanical stress.

Interestingly, the nuclei of oocytes in primordial follicles at the edge of the ovaries have been observed to rotate (Figure 4c) (Nagamatsu et al., 2019). The kinetics of nuclear rotation in the oocytes was similar to that observed in other cell types. Namely, the nuclear rotation in the oocytes was induced by mechanical pressure, as the rotation ceased in the collagenase‐treated ovaries, and resumed upon the addition of exogenous pressure to the ovaries. Compared to the obvious change in the conformation of migrating cells, oocytes maintain a round shape that is unlikely to generate a biased distribution of the cytoskeleton network. In addition, exogenous air pressure would exert “compression stress” on oocytes, but this would also be unlikely to change the cell shape. We found that nuclear rotation was observed only in the oocytes in the primordial follicles at the edge of the ovary, whereas those inside the ovaries were stationary (Figure 4c). Although the reason for these different behaviors is still elusive, nuclear rotation may be driven in the oocytes under a specific condition, such as a biased distribution of ECM at the edge of the ovary, and the compression may change the threshold of nuclear rotation.

Much as in other cell types, motor proteins play an essential role in the nuclear rotation of oocytes. When ovaries were cultured with Ciliobrevin D, a cytoplasmic dynein inhibitor, nuclear rotation completely ceased in the oocytes (Nagamatsu et al., 2019). Importantly, the dynein inhibition induced translocation of FOXO3 into the cytoplasm, followed by follicular activation. This suggests that a dynein‐mediated action, perhaps nuclear rotation itself, has an important role in the dormancy of oocytes. As described above, microtubules with dynein proteins connect the nuclear membrane to the cell membrane, which constitutively controls the tension between these membranes. Compression might alter the tension, which would affect the flexibility of the nucleus of the oocytes. This possibility will be evaluated in a future work. Nevertheless, these observations indicate that the nuclear rotation is one of the outcomes of mechanical stress that contribute to dormancy of the oocytes in the primordial follicles.

## 6 A POSSIBLE HIERARCHY OF FACTOR DETERMINING THE GEOGRAPHY OF THE OVARY

**Figure 5:**
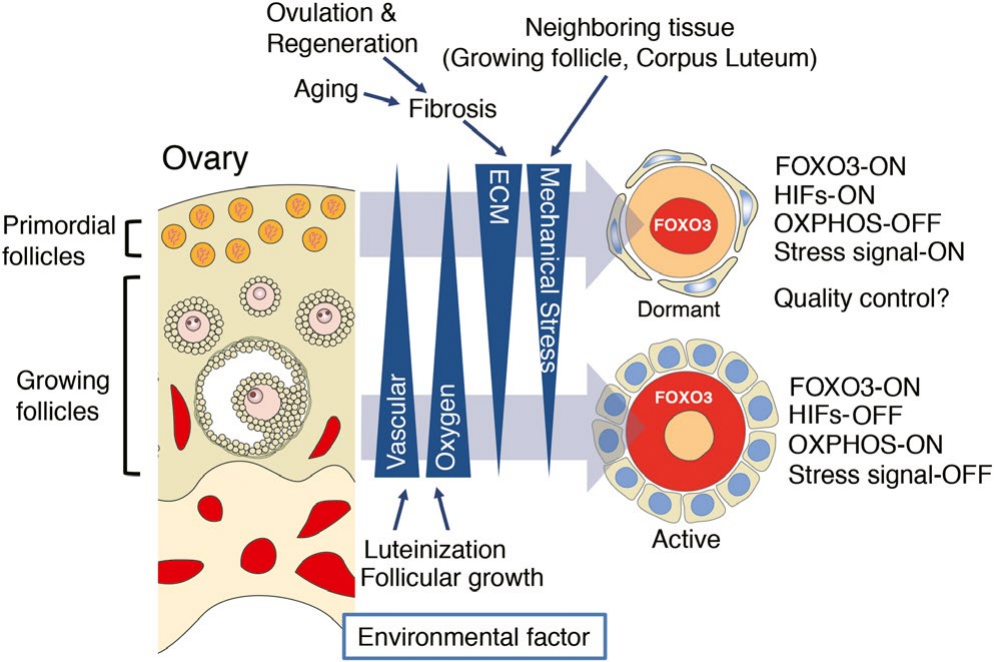
A model of ovarian environment that controls follicular development. During ovarian organogenesis, gradients of oxygen and ECM are established in the ovary. The ovarian surface is far from the vascular network in the medulla, indicating that primordial follicles reside under a hypoxic condition. ECM is enriched in the cortical region. ECM may generate a mechanical stress that keeps oocytes dormant. These environmental factors are highly dynamic due to physiological and histological changes in the ovary, such as folliculogenesis, ovulation and aging. In response to these environmental factors, oocytes change the transcriptional network and metabolism, which may contribute to maintenance of quality of oocytes for a long period of time in the ovary

Here, we identify environmental factors responsible for maintaining the dormancy and possibly directing the localization of dormant oocytes in the ovary. Based on the findings described above, there are at least two environmental factors that exist in gradients in the ovary; one is oxygen and the other is ECM (Figure 5). Regarding oxygen supplementation, the ovarian cortex is less vascular than the ovarian medulla, indicating that oocytes in the cortex region are exposed to a hypoxic condition. Although it is technically challenging to measure precisely the concentration of oxygen at a specific region of the ovary in vivo, histological analysis showed that primordial follicles do not exist near blood vessels (Feng et al., 2017). It is known that a vascular network dynamically changes in the ovaries in association with follicular development and ovulation (Fraser, 2006). For example, angiogenesis is promoted by VEGF‐signaling activated during folliculogenesis and luteogenesis. Therefore, the local oxygen concentration is altered at every estrus cycle. Although the cortical region is relatively avascular, it is possible that oxygen supplementation from newly formed blood vessels is one of the signals that trigger follicular activation.

It is reported that ECM is relatively enriched in the cortical region, compared to the medulla region, indicating that there is a gradient of ECM in the ovary (Figure 5). Due to physiological and histological change in the ovary, this gradient is not static but rather dynamic. For example, when the corpus luteum degenerates, luteal cells are replaced with fibroblasts and macrophages, by which dense connective tissue, such as corpus albicans, is newly formed. Apart from this, aging is also a factor that promotes fibrosis (Umehara, Richards, & Shimada, 2018). It is also possible that metalloproteases from serum and/or cells locally degrade ECM in the ovary. Taken together, these findings suggest that the dynamics of the local environment are at least regulated by oxygen and ECM concentration.

## 7 REGENERATION OF FETAL VS. ADULT OVARIAN TISSUE

Since the development of iPSCs, much effort has been devoted towards reconstituting specific cell types and tissues. Although such in vitro‐differentiated tissues show gene expression and organization similar to those of actual tissues in vivo, in many cases they remain immature in key ways. For example, iPSC‐derived cardiomyocytes exhibit immature features in their ultrastructure, physiological function and metabolism (Denning et al., 2016). iPSC‐derived kidney tissues also show immature features in their histology and organ structure (Nishinakamura, 2019). A similar phenomenon has been reported in ovaries—namely, reconstituted ovaries composed of PGCLCs and E12.5 gonadal somatic cells failed to establish dormant oocytes. This indicates that the culture system cannot produce an environment sufficient for the establishment of dormancy, which is a feature of ovaries after birth. Therefore, new methods will be needed to induce maturation of the regenerative tissue.

There are several possible approaches to inducing the maturation of regenerative tissue. These include (a) adding growth factors, small chemicals and other components to the medium, (b) controlling the temperature and oxygen concentration, (c) co‐culturing with appropriate cells, (d) developing a culture device that mimics the environment in vivo, such as a 3D‐structure, and (e) physically and/or electrically stimulating the tissue. Based on our study, the oxygen concentration and mechanical stress (compression) are important for the maturation of ovarian tissue in order to construct a suitable environment for dormant oocytes. However, transcriptome analysis revealed that dormant oocytes generated by either hypoxia or compression were not completely identical to dormant oocytes in vivo. This led us to test the combined effect of the two conditions on the acquisition of dormancy in oocytes. In the meantime, it will also be important to develop a methodology to measure oxygen concentrations and mechanical stresses in the ovary in vivo.

## 8 CONCLUSION

The dormancy of oocytes is acquired under a specific environment in the ovary, which has not been successfully reconstituted in a culture system. Screening of genes that are missing in in vitro‐produced oocytes and subsequent culture experiments revealed that hypoxia and ECM‐mediated mechanical stress are important conditions in this environment. The identification of such environmental factors is not only a key to understanding the mechanisms underlying oocyte dormancy, but also crucial information for refining a reconstitution system which can properly reconstitute the tissue. Indeed, it has been suggested that a hypoxic condition may control the quality of oocytes by repressing ROS production. This may be a mechanism by which dormant oocytes ensure the transmission of quality genetic and epigenetic information as well as mitochondria to the next generation. The mechanical stress also provides a novel insight into the dormancy of the oocytes. Studies on involvement of mechanical stress in the dormancy at least partially reveal why dormant oocytes are located in a cortical region of the ovary. These findings are particularly informative for proper reconstitution of an ovarian tissue in culture. Since the maintenance of dormant oocytes is a feature of the adult ovary, the effects of mechanical stress may be relevant to tissue maturation, which is a general issue in regenerative medicine. Further investigation will reveal crosstalk between hypoxia and mechanical stress, by which the quality of oocytes may be regulated in the dormant state.

## CONFLICT OF INTEREST

All of the authors declare that there is no conflict of interest that could be perceived as prejudicing the impartiality of the research reported.
